# *Aeromonas hydrophila* Survives the Treatment of Posttraumatic Cellulitis in the Shelter of an Obscured Fish-Bone Fragment

**DOI:** 10.1155/2020/6498950

**Published:** 2020-10-23

**Authors:** Areti Ganiatsa, Constantina Gartzonika, Georgios Gaitanis, Paraskevi Voulgari, Stamatina Levidiotou-Stefanou, Ioannis D. Bassukas

**Affiliations:** ^1^Department of Skin and Venereal Diseases, Faculty of Medicine, School of Health Sciences, University of Ioannina, Ioannina, Greece; ^2^Department of Microbiology, Faculty of Medicine, School of Health Sciences, University of Ioannina, Ioannina, Greece; ^3^Rheumatology Clinic, Department of Internal Medicine, Faculty of Medicine, School of Health Sciences, University of Ioannina, Ioannina, Greece

## Abstract

Fish bone and/or spine puncture injuries can result in infection of the upper extremities with aquatic bacterial pathogens. Additionally, in such injuries, the inoculation of foreign organic material is frequent and may further complicate the clinical presentation and course of the resulting infection. We describe the case of a 45-year-old female patient with a minimal fish rostrum puncture trauma acquired during preparation of fresh fish meal, which resulted in a galloping hand cellulitis. The alarming clinical presentation and the prompt response of the skin infection to clindamycin obscured the presence of inoculated fish rostrum remnants in the tissue that, three weeks later, gave rise to a foreign body granuloma, from which *Aeromonas hydrophila* was isolated. Final resolution was achieved with an additional two-week doxycycline treatment. In conclusion, the reported case highlights the potential of the accidentally implanted organic material, as are fish bones, not only to transfer uncommon pathogens but also to offer a sanctuary that favors microbial survival despite antibiotic therapy thus enabling latent or recurrent infections.

## 1. Introduction

Fish bone and/or spine puncture injuries can result in infection of the upper extremities with aquatic bacterial pathogens [1–3]. Additionally, in such injuries, the inoculation of a foreign organic material is frequent and may further complicate the clinical presentation and course of the resulting infection [1].

Herein, we describe a case of a fish rostrum puncture that resulted in a galloping hand cellulitis. The alarming clinical presentation obscured the presence of the fish rostrum remnants in the tissue that, three weeks later, gave rise to a foreign body granuloma, from which *Aeromonas hydrophila* was isolated. This case is a reminder that, in any fish bone injury, the insertion of a foreign body should always be considered and, if possible, localized and timely removed.

## 2. Case Report

An otherwise healthy, 45-year-old female presented with a painful, rapidly expanding erythematous edema of her right hand that developed within 5 hours after a puncture trauma during preparation of a swordfish for cooking. The edema involved the right hand and forearm and restricted hand function. No fever or palpable lymph nodes were present, and the puncture point was not visible. White blood cell counts, erythrocyte sedimentation rate, and C-reactive protein levels were within normal limits. No pathogens were isolated from superficial swab cultures. Cellulitis was diagnosed, tetanus prophylaxis with tetanus immunoglobulin (Tetagam®) was given, and the patient was treated with clindamycin (i.v. 600 mg t.i.d.) and ciprofloxacin (i.v. 400 mg b.i.d.). A three-day course of oral glucocorticosteroids (32 mg methylprednisolone q.d.) was added to reduce the excessive edema. The clinical condition improved promptly with substantial reduction of the edema after the first 24 hours of treatment, and since no pathogens were isolated from the superficial lesional skin swabs, the treatment was not revised. The patient significantly improved during a 7-day hospitalization and was discharged with the instruction to continue treatment with oral clindamycin (300 mg t.i.d.) for additional 2 weeks.

**Figure 1:**
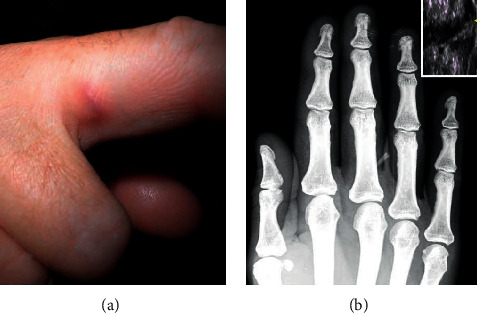
(a) Skin lesion at the 2nd admission. (b) X-ray film of the right hand demonstrating a wedge-formed radio-opaque foreign body in the soft tissue of the lateral aspect of the 1st phalanx of the middle finger. Inset: ultrasound detail of the foreign body insertion site.

Three weeks later, the patient returned with a tender, slow-growing, 8 mm large inflammatory nodule in the medial aspect of the first phalanx of the right middle finger (Figure 1(a)). The patient was otherwise healthy, and no further symptoms were reported. Subsequently, she was re-evaluated for nontuberculous mycobacteria infection and/or an evolving foreign body granuloma. Blood tests were within normal range, and the tuberculin skin reaction was negative. A tissue probe was transferred in anaerobic transport medium and cultured under aerobic and anaerobic conditions as well as in the solid and liquid medium for mycobacteria. The only pathogen isolated in tissue cultures was *A*. *hydrophila*. Identification and susceptibility testing were performed using conventional microbiological methods and the Vitek 2 system (bioMerieux, France). According to the susceptibility pattern, *A*. *hydrophila* was mainly sensitive to third-generation cephalosporins, aminoglycosides, and fluoroquinolones and to trimethoprim-sulfamethoxazole. Ultrasound and X-ray imaging of the area revealed the presence of a foreign body located at the site of the emerging granuloma, immediately beneath the skin (Figure 1(b)). The foreign body was removed; macroscopically, it was consistent with fish bone remnants. The patient was subsequently treated with oral ciprofloxacin for two weeks. The inflammation regressed quickly, and the patient had no evidence of infection at the 6-month follow-up examination.

## 3. Discussion

The rapidly progressing course of the present hand cellulitis coupled with the reported fish puncture injury raised the suspicion of a polymicrobial infection with the participation of marine pathogens, particularly *Vibrio vulnificus*, a species known to cause rapidly evolving skin and soft-tissue infections (SSTIs) [4–6]. However, the initial working diagnosis had to be modified after the development of a foreign body reaction and the following isolation of *A*. *hydrophila* from it. In retrospect, we suggest that *A*. *hydrophila*, if not the exclusive pathogen, was at least an important copathogen in a polymicrobial traumatic hand cellulitis. This hypothesis complies with the following clinical features of the present case: (1) Rapid response of the cellulitis to the initial combination treatment with intravenous ciprofloxacin and clindamycin. (2) Persistence of the microorganism in the relatively inaccessible to an antimicrobial sanctuary of the fish-bone fragments and its isolation after the development of the purulent granuloma. It is known that biofilm formation is facilitated by the presence of a foreign material and significantly increases the antibiotic resistance of microbes in the tissues [7]. The employment of the fish bone as a scaffold for the development of a biofilm was probably the main strategy of *A*. *hydrophila* to escape eradication during the first antibiotic course in this patient. (3) Unequivocal cure after the removal of the foreign body and an additional course of antibiotics. (4) *A*. *hydrophila* is frequently isolated from sites of polymicrobial hand infections, and most of the SSTIs with similar as the above clinical features are of polymicrobial nature [8, 9]. However, an accidental reinfestation of the patient with the same pathogen *A*. *hydrophila* cannot be fully excluded; this has been already described in the literature to explain some cases of hand infections due to *V*. *vulnificus* [4].

*Aeromonas* species are facultatively anaerobic, Gram-negative, rod-shaped bacteria that are ubiquitous in aquatic environments [3, 8]. They are frequently isolated from fresh or brackish water (rivers, lakes, ponds, and estuaries) including aquatic habitants, sewage, soil, and tap water, particularly during the warmer months of the year [3, 8]. In addition, different motile *Aeromonas* species, including *A*. *hydrophila*, are important fish pathogens [10]. In humans, they are opportunistic pathogens mostly implicated in gastroenteritis cases from the ingestion of inadequately processed contaminated food and SSTI [8, 11]. Skin trauma and exposure to environmental water appear to be the principal factors involved in the pathogenesis of SSTI due to *A*. *hydrophila* [3, 9]. In particular, *A*. *hydrophila* is regarded as the most common single cause of SSTI associated with fresh water injuries [12] that include simple abrasions and puncture wounds to less common yet exotic scenarios such as propeller accidents and alligator bites [3, 8]. Likewise, the resulting clinical spectrum ranges from cellulitis and localized skin and soft-tissue nodules and abscesses to alarming rapid spread of the pathogen into deeper layers of the skin and subcutaneous tissues [8, 11]. Therefore, the development of cellulitis with fulminant progression within a few hours and the anamnesis of an eliciting skin trauma in the aquatic environment or during handling fresh fish should alert the clinician for a possible *A*. *hydrophila* infection [8, 13]. In such cases, the prompt onset of antibiosis is essential in order to stop the invasive course of the infection, which may quickly progress into ecthyma gangrenosum, necrotizing fasciitis with severe myonecrosis of the adjacent muscles, osteomyelitis, and septicemia [3, 8, 9]. These latter, more severe complications are predominantly seen in patients with certain predisposing factors such as liver disease or malignancy [3, 9]. Surgical decompression with incision, drainage, and debridement may be required in order to prevent extensive tissue necrosis. In our present case, no surgical intervention was performed at initial presentation; the timely onset of a short systemic methylprednisolone course has probably induced the prompt reversion of the galloping edema. However, it may have also contributed to the initial masquerading of the presence of the foreign body [14].

The present case illustrates the persistence of the skin infection as the result of the omitted search for a foreign body during the initial patient's consultation. Therefore, the history of such a trauma should always raise concern of possible implantation of a slowly degradable organic material that could favor the survival of inoculated pathogens and enable persistent or latent-recurrent infections [1]. However, it is worth noting that routine X-ray film examinations are not always reliable to detect inserted fish tissue fragments as their radiographic visibility decisively depends on the specific composition of the foreign material [15]. Using a soft-tissue technique will probably improve the efficacy of the latter study. Additional imaging studies, such as sonography or MRI, may be useful in cases of increased suspicion and negative radiologic findings.

In conclusion, community-acquired cellulitis following skin trauma in contact with environmental water or aquatic organisms should alert the clinician for the possibility of infection with uncommon marine pathogens including *A*. *hydrophila* [8, 16].
